# West Nile virus outbreak in Pakistan: Another potential public health threat in the pipeline?

**DOI:** 10.1016/j.amsu.2022.103663

**Published:** 2022-04-22

**Authors:** Summaiyya Waseem, Taha Gul Shaikh, Syed Hassan Ahmed, Rana Muhammad Usama, Muhammad Sohaib Asghar, Muhammad Junaid Tahir

**Affiliations:** a Dow University of Health Sciences, 74200, Karachi, Pakistan; b Lahore General Hospital, Lahore, 54000, Pakistan; c Dow University of Health Sciences–Ojha Campus, 75300, Karachi, Pakistan

**Keywords:** Arbovirus, Virus, West Nile fever, Infection, Global health, Epidemic

## Abstract

West Nile virus (WNV), a single-stranded RNA virus belonging to the family of Flaviviridae, is an arbovirus transmitted to humans predominantly by mosquito bites. It exhibits a wide range of clinical findings ranging from asymptomatic presentation to severe several neurological disorders. WNV has afflicted several countries around the globe including Pakistan. News of yet another outbreak in the country by WNV is circulating again. Concerned authorities should act vigilantly before the endemic takes over completely and bring down the already bereaving healthcare of Pakistan.

Dear Editor,

Discovered and named after the West Nile district of Uganda, West Nile virus (WNV) is a positive-strand RNA virus replicating via RNA-dependent RNA polymerase cycle [1]. It belongs to Japanese encephalitis serocomplex and has five phenotypical lineages, however only lineages 1 and 2 are considered human pathognomonic [2]. As a mosquito-borne infection, its main transmission is via Culex mosquitoes, which particularly infect mammals including birds and humans. However, there is evidence of infections via organ transplant, blood transfusions, breast milk, and transplacental transmission [3].

The clinical manifestation following a WNV infection spans a wide range, with about 80% population remaining asymptomatic. Mild symptomatology includes systemic febrile illness, also known as West Nile Fever (WNF). The jeopardizing symptoms are seen in less than 1% population and develop when the virus invades the central nervous system (CNS), thereby leading to meningitis, encephalitis, or acute poliomyelitis-like syndrome [4]. The severe form can lead to threatening complications including paralysis, vision loss, and even death. Diagnosis of the virus is dependent on serological testing and the presence of IgM antibody in serum indicates a positive infection. The cerebrospinal fluid sample is collected as well, presence in which suggests CNS invasion. In immunosuppressed demographics, delayed antibody production can lead to a false negative, hence nucleic acid amplification tests may be performed [2]. Currently, there is no specific vaccine for the virus, and treatment is mainly supportive.

The virus has been endemic throughout the world including Israel, Africa, Asia, the Caribbean, Greece, Romania, Russia, and the United States of America (USA) [5]. Among the Mediterranean region, Pakistan has been under its pounce with WNV RNA detected in 33.3% of the population [6]. Another report by Zohaib et al. found seropositivity for the virus in 65% of the samples collected [7]. Serological testing and detection of WNV in the local population of Pakistan dates back to as early as 1982 [8], while the most recent outbreak was recorded in 2015–16 [9]. Currently, reports of the WNV outbreak are circulating again. According to an infectious diseases specialist from a renowned hospital in the country, the clinical presentation of patients in hospital along with a massive number of crows' and birds’ death, highly suggests that the country is facing an outbreak of WNV once again. However, due to the lack of testing facilities, there is a long way to a confirmatory conclusion [10]. There can be multiple reasons for the potential outbreak, however, previous literature cites that lack of adequate testing centers, poor sanitation, inadequate lifestyles, absent surveillance system, flooding, geopolitical instability, and easy access to neighboring countries via the construction of highways have made Pakistan, more prone to arboviral infection, primarily Crimean-Congo hemorrhagic fever virus (CCHFV), WNV, Chikungunya virus (CHIKV), and Dengue viruses (DENV) [9].

To ensure that the country does not undergo another healthcare hazard, all precautionary measurements must be employed. Government must allocate more funds to the healthcare department. Currently, Pakistan lacks testing services for WNV except for a few institutes [10]. Increasing testing rates will help in establishing a conclusion if there is really an endemic on the way. Moreover, a highly proficient plaque reduction assay should be introduced to differentiate between different flavivirus as most of them present with the same clinical spectrum. As the major transmission source of WNV remains mosquitoes, special emphasis must be laid on introducing high sanitation facilities across the country, ensuring removal of stagnant waters from streets, and purification to reduce mosquito survival. This will also help reduce the other arboviral infections alongside. Since there has been evidence of its transmission via blood transfusions, introducing tests during the transfusion process may reduce the horizontal spread. For controlling vertical transmission, maternity testing should be made compulsory.

Public awareness programs should be run throughout the country, educating masses on personal hygiene, use of mosquito repellants, and electrical devices. Database systems for proper reporting of any case must be well-developed and the cases should be followed-up. Lastly, research programs should be devised that will work on actively controlling the transmissions via investigating every possible route. As a lower-middle-income country, still recovering from coronavirus disease 2019 (COVID-19) blackout and dengue outbreaks, a potential threat of WNV can lead to a disastrous setback, thereby further depriving the healthcare economy of Pakistan. Hence it is imperative that the concerned authorities take immediate actions or WNV can be the next gigantic public health threat for the country.

## Ethical approval

Not required.

## Sources of funding

None.

## Author contribution

S·W and T.G.S conceived the idea, S·W, T.G.S, S·H.A, and R.M.U retrieved the data, did write up of letter, and finally, M.J.T and M.S.A reviewed and provided inputs. All authors approved the final version of the manuscript.

## Registration of research

1 Name of the registry: Not required.
2. Unique Identifying number or registration ID: N/A
3. Hyperlink to your specific registration (must be publicly accessible and will be checked):

## Guarantor

Muhammad Sohaib Asghar.

## Financial disclosure

No financial support was provided relevant to his article.

## Declaration of competing interest

All authors report no conflict of interest relevant to his article.
